# A Novel Application of Recycled Ultrafiltration Membranes in an Aerobic Membrane Bioreactor (aMBR): A Proof-of-Concept Study

**DOI:** 10.3390/membranes12020218

**Published:** 2022-02-14

**Authors:** Laura Rodríguez-Sáez, Sotiris I. Patsios, Jorge Senán-Salinas, Junkal Landaburu-Aguirre, Serena Molina, Eloy García-Calvo

**Affiliations:** 1 IMDEA Water Institute, Av. Punto Com, 2, Alcalá de Henares, 28805 Madrid, Spain; jorge.senan@uvic.cat (J.S.-S.); junkal.landaburu@imdea.org (J.L.-A.); serena.molina@imdea.org (S.M.); eloy.garcia@imdea.org (E.G.-C.); 2 Chemical Engineering Department, Alcalá University, Alcalá de Henares, 28805 Madrid, Spain; 3 Laboratory of Natural Resources and Renewable Energies, Chemical Process & Energy Resources Institute (CPERI), Centre for Research and Technology-Hellas (CERTH), GR Thermi, 57001 Thessaloniki, Greece; patsios@certh.gr

**Keywords:** membrane bioreactor (MBR), recycled ultrafiltration membrane, circular economy, recycling, cost analysis

## Abstract

The use of recycled ultrafiltration (r-UF) membranes, originating from end-of-life reverse osmosis membranes, as submerged flat-sheet membranes in an aerobic membrane bioreactor (aMBR) system is described herein for the first time. A feasibility study of this new approach was performed in a laboratory-scale aMBR system. The r-UF membrane performance was evaluated in terms of permeability, fouling behavior, and permeate quality using a widely used commercial flat sheet microfiltration membrane (c-MF) as a reference. Tests were conducted under steady-flux operation (at 12 and 14 L·m^−2^·h^−1^) and a variable trans-membrane pressure. Synthetic wastewater simulating urban wastewater characteristics with approx. 0.4–0.5 g/L COD concentration was used as the feed. The obtained results showed that the rejection performance of the r-UF membrane was similar to the performance of the commercial flat sheet microfiltration membrane (c-MF) under comparable operating conditions. Moreover, concerning fouling behavior, the r-UF membrane exhibited higher fouling resistance compared with the c-MF membrane, although the permeability decline rate was lower. Both membranes had comparable fouling mechanisms behavior, with cake layer fouling resistance accounting for approx. 60% of the total fouling resistance. Finally, a preliminary economic assessment pointed out the potential competitiveness of using r-UF membranes for aMBRs (5.9–10.9 EUR·m^−2^) and the scaling-up challenges toward industrial applications.

## 1. Introduction

During the last few decades, membrane bioreactors (MBRs) for wastewater treatment have gained increasing significance. The installed capacity in 2019 was estimated at over 2 × 10^7^ m^3^ per day, including medium- and high-capacity treatment plants [1]. The trend is also positive when economic data are considered. The global market of MBR reached EUR 2.5 billion in 2019 and it is expected to reach EUR 3.5 billion by 2024 [2]. The main reason for this economic growth is the notable advantages of MBRs compared with the conventional activated sludge (CAS) process. The use of membranes for treated effluent separation allows for the MBR to operate at higher volumetric loading rates than CAS systems, leading also to smaller space requirements for the facilities. In addition, an MBR provides higher removal efficiency regarding pollutants, nutrients, microorganisms, and suspended solids due to the use of membranes.

Despite these advantages, MBR processes still face some difficulties that need to be overcome. Specifically, increased energy consumption and membrane fouling, which leads to significant chemicals consumption and early membrane replacement, substantially increase MBR operating expenses (OPEX) [3]. According to Iglesias et al., the replacement cost of the membranes can contribute between 0.02 and 0.04 EUR per m^3^ of produced effluent (assuming a membrane lifespan of 8–10 years), which accounts for approximately 10–12% of the OPEX [4]. This membrane-related cost is even higher if energy consumption for aeration and chemicals used during membrane cleaning are considered. Moreover, the service life of MBR membranes is affected by the frequency of the cleaning cycles, ultimately resulting in membrane replacement with the consequent additional costs. This is one of the reasons that there is a significant amount of ongoing research focused on the assessment of novel low-cost membranes and membrane surface modification methods for fouling prevention and mitigation (e.g., [5,6]).

Senán-Salinas et al. reported that it would be possible to obtain recycled modules with a price range of 45–100 EUR per module, depending on the recycling strategy [7]. Even though the unitary cost for a commercial UF membrane module could be affected by the peculiarities of every acquisition’s transaction, it could be considered that the pristine commercial module prices are between 400–800 EUR per module [7,8]. Therefore, in view of the above data, the use of recycled membranes could offer a competitive alternative to reduce the membrane replacement cost in the MBR process.

Currently, waste-related legislation in most countries still follows the traditional concept of a linear economy. Consequently, most end-of-life (EoL) membranes are disposed of in landfills without considering other management alternatives, in contrast with the circular economy principles set by the European Commission [9]. In this context, research studies are reported on implementing novel reverse osmosis (RO) membrane recycling/reuse strategies, following the circular economy approach [10,11,12,13,14]. Until now, the research and development of such recycled membranes have focused on verifying their feasibility and suitability for diverse processes, such as (i) direct reuse as RO membranes [10], (ii) direct recycling of EoL RO membranes as nanofiltration (NF) and ultrafiltration (UF) membranes [11], (iii) use as a support for biofilm-membrane reactors [12], and (iv) indirect recycling as membrane support for ion exchange electrodialysis membranes [13]. Recycling processes have also been tested at a pilot scale and validated in water treatment facilities [14]. However, to the authors’ best knowledge, the recycling of RO membranes and their application on MBR systems have never been tested.

The present work is a proof-of-concept study that aimed to evaluate the feasibility of using recycled ultrafiltration (r-UF) membranes obtained from EoL RO membranes as submerged flat-sheet membranes in an aerobic MBR (aMBR) system. The process performance of the r-UF membranes was evaluated in terms of the (i) membrane permeability, (ii) resulting permeate quality, and (iii) membrane fouling behavior. Furthermore, a preliminary cost analysis of the use of r-UF membranes is discussed to provide insight into the economic feasibility of the proposed application.

## 2. Materials and Methods

### 2.1. Chemicals

The chemicals used for the preparation of synthetic urban wastewater were glucose (C_6_H_12_O_6_) D(+) glucose anhydrous, extra pure, Ph Eur, BP, USP (Sigma-Aldrich; Baden-Württemberg, Germany); meat peptone (Sigma-Aldrich; Baden-Württemberg, Germany); urea (Urea, ACS reagent grade, Sigma-Aldrich; Baden-Württemberg, Germany); sodium chloride (NaCl reagent grade, ACS, ISO, Reag. Ph Eur, Sigma-Aldrich; Baden-Württemberg, Germany); sodium bicarbonate (NaHCO_3_, extra pure, Pharmpure^®^, Ph Eur, BP, USP, Sigma-Aldrich; Baden-Württemberg, Germany); di-potassium hydrogen phosphate anhydrous (K_2_HPO_4_ for analysis, ExpertQ^®^, ACS, Reag. Ph Eur, Sigma-Aldrich; Baden-Württemberg, Germany); calcium chloride dihydrate (CaCl_2_·2H_2_O powder, for analysis, ExpertQ^®^, ACS, Sigma-Aldrich; Baden-Württemberg, Germany); magnesium sulfate heptahydrate (MgSO_4_·7H_2_O for analysis, ExpertQ^®^, ACS, Reag. Ph Eur Sigma-Aldrich, Baden-Württemberg, Germany); and iron (III) chloride hexahydrate, (FeCl_3_·6H_2_O ACS reagent 97%, Sigma Aldrich, Baden-Württemberg, Germany). The chemicals used for the membrane recycling process and membrane cleaning were sodium hypochlorite (NaOCl 10% *w*/*v*, Scharlab; Barcelona, Spain) and ethanol (96% EPR Ph.Eur. Sigma-Aldrich; Baden-Württemberg, Germany). Samples and solutions were prepared using Milli-Q water.

### 2.2. Membranes: Description and Characterization

The performance of an r-UF membrane was evaluated in terms of the (i) membrane permeability, (ii) permeate quality, and (iii) membrane fouling behavior. A c-MF membrane (Table 1), widely used in MBR systems, was also employed as a reference membrane to set the performance benchmarks for the evaluation of the r-UF membrane performance. The c-MF was selected for its wide-ranging application in MBR plants in Spain [4].

**Figure 1:**
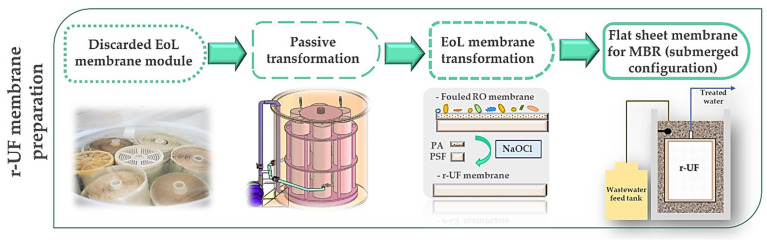
r-UF membrane preparation flow chart.

As Figure 1 shows, the r-UF membranes were obtained by eliminating the polyamide (PA) layer of EoL RO membranes by means of exposure to a NaOCl dose of 800,000 ppm·h, according to the procedure proposed by García-Pacheco et al. [15]. Membrane transformation was conducted on the whole module at a pilot scale [14]. The recycling process was performed by conducting a passive transformation by immersing the entire modules into the NaOCl solution to chemical attack the membrane surface to eliminate the polyamide layer. Then, the module was disassembled to obtain the membrane samples used in the present study. Coupons (0.06 m^2^ area) of the r-UF membrane (12 nm nominal pore size) were used in this study

### 2.3. Experimental Set-Up

The experiments were performed in a lab-scale aerobic MBR system that allowed for continuous operation. The MBR tank volume was approx. 18 L and a flat sheet membrane module with an effective membrane area of 0.11 m^2^ in a submerged configuration were employed. A piston pump (Fluid Metering Inc.; Syosset, NY, USA) was used for constant flux operation, whereas a pressure transducer recorded the temporal evolution of the TMP. A pH meter with an integrated temperature sensor (713-type pH meter, Metrohm Ltd.; Herisau, Switzerland) was used to monitor the pH and temperature of the bioreactor. The laboratory-scale aMBR was built and set in operation at the Natural Resources and Renewable Energies Laboratory (NRRE) of the Chemical Processes and Energy Resources Institute (CPERI/CERTH; Thessaloniki, Greece). Details of the construction and characteristics of the aMBR unit can be found elsewhere [18].

The membranes were tested in the aMBR unit using synthetic wastewater (SWW) simulating urban wastewater, with an approximate COD concentration of 0.4–0.5 g/L. The synthetic wastewater feed was selected, instead of real municipal wastewater, to minimize the fluctuations in the parameters of the feed and render the operating conditions of the aMBR steady and reproducible regarding its feed characteristics. The feed stream was made by diluting a 50-times-concentrated cSWW with tap water. To avoid early contamination and spoilage, concentrated SWW was pasteurized by placing it in an oven at 50–60 °C for 5–6 h. The concentrated SWW composition was as follows: 300 mg/L C_6_H_12_O_6_, 100 mg/L peptone, 30 g/L CH_4_N_2_, 28 mg/L K_2_HPO_4_, 7 mg/L NaCl, 4 g/L CaCl_2_, 2 mg/L MgSO_4_, 2 mg/L FeCl_3_·6H_2_O, and 150 mg/L NaHCO_3_. Initial sludge inoculum was obtained from the Municipal Wastewater Treatment Plant of Thessaloniki. To assess the MBR performance, the trans-membrane pressure (TMP), pH, and temperature data were monitored and stored daily. The MBR feed and permeate were analyzed twice a week, together with the characterization of the mixed liquor properties. The mixed liquor suspended solids (MLSS), dissolved oxygen (DO), biochemical oxygen demand (BOD_5_), and chemical oxygen demand (COD) were determined based on APHA Standard Methods procedures [19]. TP and TN were determined colorimetrically (UV-1700 Spectrophotometer, Shimadzu Co.; Kyoto, Japan) based on DIN 38405 D9 (N-NO3) and DIN EN ISO 6878 (P-PO4) methods, respectively, after sample digestion using sulfuric acid/peroxydisulfate and alkaline potassium persulfate. TOC was measured using a TOC analyzer (TOC-5000A, Shimadzu Co.; Kyoto, Japan). DO concentration in the bioreactor was measured by an oxygen probe (Z921, Consort). MLSS were measured via filtration on a Whatman GF/A microfiber glass filter (1.6 μm nominal pore size).

A steady-flux (*J*, L·m^−2^·h^−1^) operation and variable TMP were selected to evaluate the membrane filtration performance. The steady-flux operation was achieved by employing a positive displacement (piston) pump (FMI—piston metering pumps), which can retain a steady volumetric flow rate, regardless of the pumping pressure (i.e., TMP). Two different flux values were employed for each membrane, i.e., 12 and 14 L·m^−2^·h^−1^, to evaluate the performance of the r-UF membrane under at least two different filtration conditions to increase the validity of the drawn conclusions. These flux values were lower compared with the flux values of commercial MBR systems (i.e., 20–30 L·m^−2^·h^−1^) to avoid operation close to the critical flux values. The critical flux value is defined as the highest initial flux where the TMP values remain rather stable [20], and it is suggested that submerged membranes of MBR systems should operate in the subcritical flux region [21]. Membrane operation was set on cycles of 8 min of suction followed by 2 min of relaxation. The laboratory-scale aMBR unit was operated at a hydraulic retention time (HRT) of 7 h. Except for the samples necessary for analyses and monitoring, no biomass was wasted from the reactor during the operation for days 1–25 (resulting in a sludge retention time SRT = ∞), whereas for days 26–41, 60 mL/d of mixed liquor (SRT = 233 days) were wasted. Regular measurements of pH, EC, and DO and MLSS concentrations were performed during the whole 41-day experiment to ascertain that the MBR operated under the same operating conditions, achieving pseudo-steady-state conditions. The average values and the SD of the aforementioned measurements were as follows: pH = 7.83 ± 0.18, EC = 942.5 ± 42.4 µS/cm, DO = 1.29 ± 0.24 mg/L, and MLSS = 5.06 ± 0.96 g/L. The low SD of the measurements denoted that the MBR operated under pseudo-steady-state conditions. The permeability decline rate (k) was estimated for different periods with linear regression models. The basic package of R software v.4 was used for the static assessment [22].

The initial experimental goal for each operating stage was to operate under the selected operating parameters for at least a week (7 days). However, after the first operating stage Ia, the duration of the following stages was increased to gather more data (e.g., for stage Ib) and/or to further study the sudden TMP increase between days 5 and 6 during stage IIb). Therefore, after a start-up period of 20 days, when the biomass was acclimatized to the operating conditions, the laboratory-scale aMBR plant operated for a total period of 41 days. During the first operating period (period I), the c-MF membrane was used, i.e., (Ia) c-MF (7 days, 12 L·m^−2^·h^−1^) and (Ib) c-MF (15 days, 14 L·m^−2^·h^−1^); whereas, during the second operating period (period II), the r-UF membrane was used, i.e., (IIa) r-UF (10 days, 12 L·m^−2^·h^−1^) and (IIb) r-UF (9 days, 14 L·m^−2^·h^−1^). The c-MF and the r-UF were meticulously mechanically cleaned between stages Ia and Ib, and between stages IIa and IIb, respectively. For the mechanical cleaning, membranes were first rinsed with tap water. Then, the membranes were, again, rinsed with tap water for one minute on each side. Then, using a wet sponge each side was vertically and horizontally cleaned. Finally, the membranes were immersed in a tank with tap water with aeration for 5 more minutes.

### 2.4. Membrane Fouling Analysis

#### Membrane Resistance Analysis

**Figure 2:**
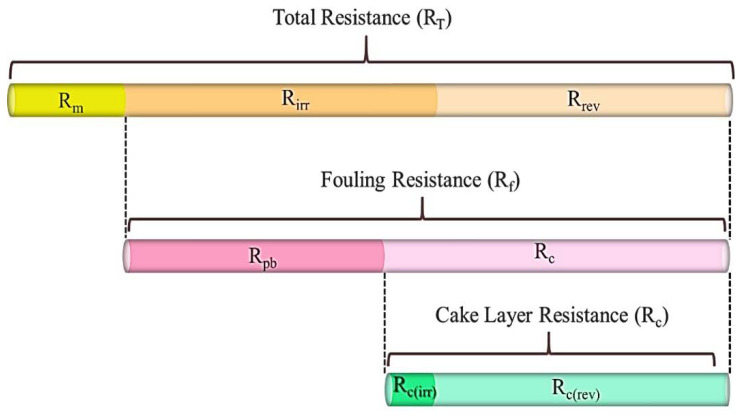
The role of fouling mechanisms in a membrane bioreactor. Modified from Di Bella et al. by specifying the two types of cake layer resistances [24].

Membrane fouling at the end of each membrane operating period (i.e., after stages Ib and IIb) was analyzed using a resistance-in-series model proposed by Di Bella et al. to assess the relative importance of pore blocking and cake layer formation on both membranes [23]. A schematic representation of the different resistances to permeation affecting the membrane performance can be seen in Figure 2.

The various filtration resistances during MBR operation can be described using Darcy’s law (Equation (1)), as follows:

(1) $$R=\frac{\mathrm{TMP}}{\mu \times J}$$

Here, R is the resistance to permeation (m^−1^), TMP is the trans-membrane pressure (Pa), µ (Pa·s) is the permeate dynamic viscosity (water viscosity at 20 °C), and J is the permeate flux (m^3^·m^−2^·s^−1^). Further, the membrane resistance R_m_ (m^−1^) is the original resistance that a pristine membrane presents during clean water filtration (Equation (2)):

(2) $$R_{m}=\frac{{\mathrm{TMP}}_{H2O}}{\mu \times J}$$

The total resistance, R_T_ (m^−1^), is defined as the sum of three different resistances at the end of each membrane operating period Ib and IIb (Equation (3)):

(3) $$R_{T}=R_{m}+R_{c\;\left(\mathrm{rev}\right)}+R_{c\left(\mathrm{ir}\right)}+R_{\mathrm{pb}}$$

where R_c (rev)_ (m^−1^) represents the reversible fraction of the cake layer resistance that is removable during relaxation or backwashing. R_c(ir)_ (m^−1^) corresponds to the irreversible fraction of the cake layer resistance that cannot be removed during relaxation or backwashing. R_pb_ indicates the fraction of the fouling affecting the membrane pores. Fouling resistance (R_f_ (m^−1^)) is defined as the total resistance due to fouling, excluding the membrane resistance R_m_ (Equation (4)):

(4) $$R_{f\;}{=R}_{c\left(\mathrm{rev}\right)}+R_{c\left(\mathrm{ir}\right)}+R_{\mathrm{pb}}$$

### 2.5. Preliminary Economic Assessment

**Figure 3:**
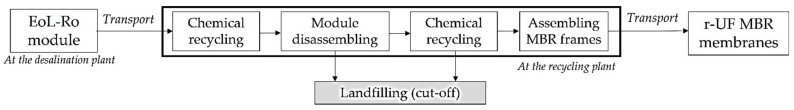
System boundaries of the economic analysis of r-UF membrane production process.

To evaluate the economic potential of recycling EoL RO membranes to MBR submerged flat-sheet UF membranes, the cost of producing r-UF MBR membranes (EUR·m^2^) was analyzed. Figure 3 shows the processes considered (i.e., the system boundaries) when conducting the cost analysis of the EoL RO recycling into r-UF MBR membranes.

The costs of EoL-RO collection and the transformation into UF were adopted from Senán-Salinas et al. [7]. The module disassembling was analyzed in Lawler et al. [25]. Finally, the adaptation of EoL-RO membrane sheets into MBR flat sheets frames was conducted by considering five different commercial and standardized MBR frames [26].

## 3. Results and Discussions

### 3.1. Characterization of the Studied Membranes: Permeability and Pore Size

**Figure 4:**
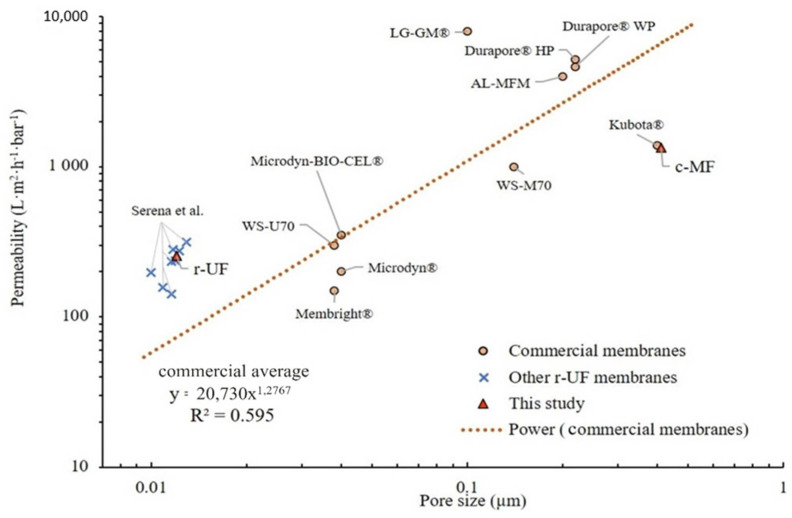
Membrane permeability for clean water according to pore size for commercial membranes and the membrane used in the present study according to Judd et al. and Molina et al. [15,26].

Membrane permeability and pore size are two of the main membrane properties that affect membrane performance and the technological niche of a UF membrane for an aMBR. To illustrate the state of the art of commercial membranes and identify the technological position of the studied r-UF membranes, Figure 4 summarizes the values of the clean water permeability versus the nominal pore size of the commercial membranes and the recycled ones.

The obtained permeability (L; L·m^−2^·h^−1^·bar^−1^) for the r-UF membrane was 255 ± 4 L·m^−2^·h^−1^·bar^−1^. This permeability value is in accordance with the average permeability range of various UF commercial membranes (i.e., 200−300 L·m^−2^·h^−1^·bar^−1^) (Figure 3). Furthermore, the results summarized in Figure 4 provide evidence of the strong non-linear relationship (R^2^ = 0.595) between the pore size and the permeability of UF and MF commercial flat sheet aMBR membranes. Even though the r-UF membrane presents a pore size that is much smaller than the pore size usually found among the commercial ones, the permeability values are in the same range as the UF commercial membranes (i.e., 200–300 L·m^−2^·h^−1^·bar^−1^).

### 3.2. Performance Efficiency of the Lab-Scale aMBR Unit

#### 3.2.1. Permeate Quality

Regarding the permeate quality, Table 2 shows the obtained results for the analyzed parameters of the permeate.

TOC, COD, and BOD_5_ average removal values for the r-UF MBR were higher than 98.9 ± 0.3% and up to 99.7 ± 0.1%. The organic matter removal efficiency of the c-MF MBR was equally high, i.e., higher than 98.2 ± 0.2%, during the whole operation. Moreover, turbidity values obtained with the r-UF membrane were very low, i.e., 0.04 ± 0.02 NTU during stage IIa and 0.01 ± 0.05 NTU during stage IIb. Taking Spanish legislation into consideration, the turbidity values obtained were consistent with the stricter requirements for treated wastewater reuse [27]. The turbidity values of the c-MF permeate were also quite low (i.e., 0.14 ± 0.01 NTU during stage Ia and 0.29 ± 0.32 NTU during stage Ib). However, the average values of the c-MF turbidity were statistically higher than the values of the r-UF. Another statistically significant difference in the average values concerning the permeate quality was the TOC concentration. Although the TOC concentration of the effluent was very low during all operating stages, the average values of the r-UF MBR permeate were slightly lower compared with the respective values of the c-MF. Overall, the permeate quality obtained using the r-UF membrane was slightly better than that obtained with the c-MF membrane. With regard to the membrane retention capacity, intrinsic properties of UF membrane (especially the lower molecular weight cut-off (MWCO)) seemed to make a difference in the retention efficiency of, e.g., dissolved solids (mainly organic macromolecules) [28]. For the r-UF membrane, the MWCO was estimated in previous studies and is considered to be around 20 kDa [29].

#### 3.2.2. Membrane Permeability Stability and Preliminary Fouling Assessment

**Figure 5:**
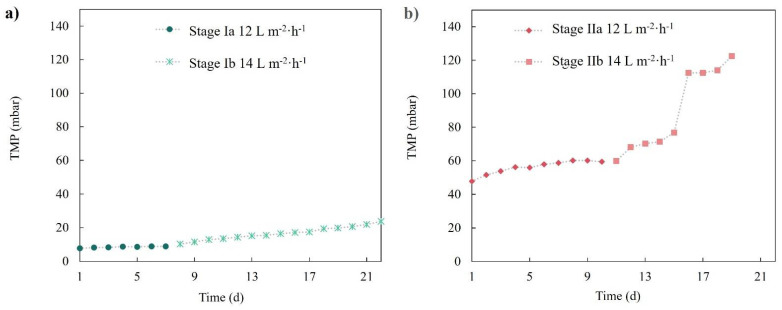
TMP temporal profile for (**a**) the c-MF membrane in stages Ia and Ib and (**b**) the r-UF membrane in stages IIa and IIb.

Figure 5 shows the transmembrane pressure evolution of both membranes (r-UF and c-MF) performing at 12 and 14 L·m^−2^·h^−1^. It was observed that the c-MF membrane barely exhibited a TMP increase when operated at 12 L·m^−2^·h^−1^ (stage Ia). Even when the flux increased to 14 L·m^−2^·h^−1^, a constant yet mild TMP increase was observed. Concerning the r-UF membrane, it was observed that its behavior changed depending on the flux value. At 12 L·m^−2^·h^−1^, the r-UF membrane presented a small TMP increase that lasted up to seven days, followed by a rather stable TMP profile for the next three days. On the other hand, the r-UF working at 14 L·m^−2^·h^−1^ showed a sharp TMP increase between days 5 and 6 that could not be attributed to a specific reason. However, after this sharp increase, the TMP seemed to stabilize.

**Figure 6:**
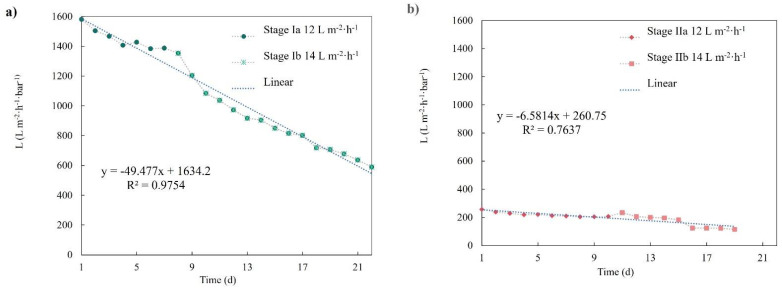
(**a**) Permeability evolution for the c-MF membrane in stages Ia and Ib and (**b**) the r-UF membrane in stages IIa and IIb.

To further assess the membrane filtration performances, the evolution of the membranes’ permeabilities was calculated. Figure 6 presents the permeability temporal profile for both membranes, together with the calculated linear permeability decline rate. The c-MF membranes presented an evident permeability decline during both experimental periods when performing at 12 and 14 L·m^−2^·h^−1^ (stages Ia and Ib). At the same time, the r-UF membrane clearly presented a milder permeability decline, especially when performing at 12 L·m^−2^·h^−1^ (stage Ia).

The permeability decline rates were also calculated for the different operating stages and are summarized in Table 3. The permeability decline values of the r-UF membrane were quite low when operating at both 12 and 14 L·m^−2^·h^−1^. The permeability decline range obtained with the r-UF membranes was similar to the commercial membrane range reported in Adham et al. for membranes with similar mean permeability (140–200 L·m^−2^·h^−1^·bar^−1^), operating in an MLSS concentration of 8–12 g·L^−1^ [30]. Therefore, upon a first look, it seems that the performance feature of the r-UF was comparable to commercial membranes, although long-term tests should be performed in future studies to confirm these results. The permeability decline rate was also slightly better than the permeability decline of the c-MF membrane under rather similar operating conditions.

A major factor affecting permeability is the fouling phenomenon, comprising the adsorption and deposition of solutes. Among other factors and membrane properties, the membrane MWCO has an important effect on the fouling phenomena. Moreover, Li et al. reported that flux decline would be more pronounced in membranes with a larger MWCO due to membranes with larger pores being more prone to pore blocking [31]. In the present work, the r-UF membrane showed a rather steady permeability decline, which, after the sixth day, appeared to be reduced or even stabilized. Due to the complex and highly variable nature of the biological sludge, the filtration performance would also depend on the particular characteristics of the sludge. This makes it necessary to take into consideration other membrane characteristics to explain the whole phenomenon. Besides the MWCO, another important factor that affects membrane behavior is surface roughness [32]. The r-UF membrane’s roughness was published by Rodríguez-Sáez et al. [17]. The r-UF membrane exhibited a roughness value (R_a_ = 4.7 ± 0.6 nm, R_q_ = 6.3 ± 1.2 nm) two orders of magnitude lower than the roughness value of the c-MF membrane. Furthermore, the obtained roughness values for the r-UF membrane were similar to the values obtained measuring commercial polysulfone UF membranes [33]. Long-term flux decline was additionally associated with the cake layer formation, where membranes with greater roughness are more prone to fouling [34]. Furthermore, it is assumed that membranes with higher hydrophilicity are less susceptible to present fouling issues [32]. The wettability of the membrane would be determined by the material that membranes are made of. According to Molina et al., the measured contact angle (CA) for r-UF membranes is over 66–68° [15]. The more hydrophilic character of the r-UF membrane, together with its lower MWCO and the lower surface roughness, probably contributed to the lower permeability decline rate compared with the c-MF membrane.

#### 3.2.3. Evaluation of Fouling Mechanisms

**Figure 7:**
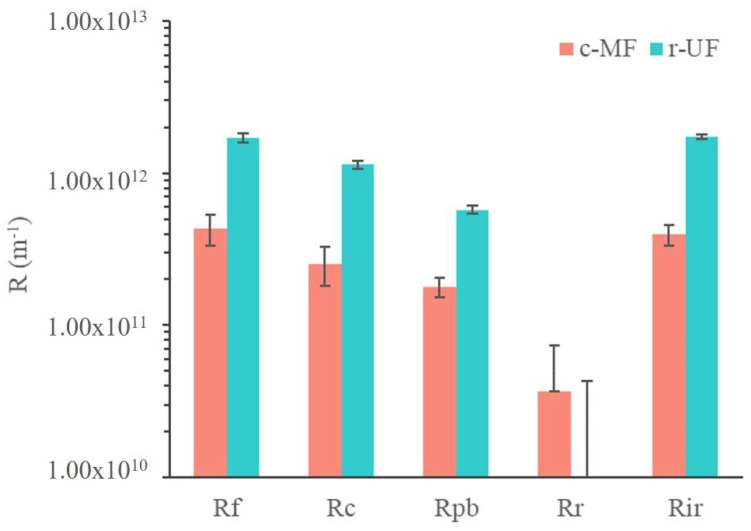
Estimated filtration resistances for the c-MF and r-UF membranes after stages Ib and IIb.

Results obtained regarding membrane fouling mechanisms are presented in Figure 7 using the resistance in series (RIS) analysis after the end of the operating period of each membrane. The presented R_c_ values include both reversible and irreversible cake layer resistance, whereas R_ir_ values comprise the resistances of the irreversible cake layer and the pore-blocking mechanism.

It is important to note that R_m_ of the r-UF membrane was, as expected, higher than that of the c-MF membrane; the R_m_ for the r-UF membrane was 1.57 ± 0.01 × 10^12^ m^−1^, and for the c-MF membrane, it was 0.27 ± 0.01 × 10^12^ m^−1^. This was due to the smaller pore size of the r-UF membrane that led to a higher resistance to water permeation. Moreover, R_m_ values for the r-UF membrane were in accordance with the R_m_ obtained for commercial polysulfone UF membranes with a similar MWCO to the r-UF one [35]. This resistance is characteristic of the intrinsic properties of the membranes.

The overall resistance to fouling was higher in the case of the r-UF membrane compared with the c-MF membrane (i.e., 1.71 ± 0.11 × 10^12^ m^−1^ compared with 0.43 ± 0.10 × 10^12^ m^−1^). The main difference was due to the resistance of the cake layer that developed on the r-UF membrane (1.13 ± 0.08 × 10^12^ m^−1^), compared with the c-MF membrane (0.25 ± 0.07 × 10^12^ m^−1^). This could be explained by the fact that the smaller pores of the r-UF membrane led to higher rejection rates, thus probably increasing the deposition of cake material. Moreover, the intrinsic higher membrane resistance resulted in higher TMP operation (even for clean membranes), thus promoting compaction of the forming cake layer. It was proposed that the cake layer in MBR is highly compressible [36], and that compaction of the (cake) fouling layer further increases the fouling resistance and renders the cake layer removal through backwashing or relaxation less effective [37].

Resistance values for R_pb_ were estimated as being rather similar for both the c-MF and r-UF membranes (i.e., 0.57 ± 0.03 × 10^12^ m^−1^ compared with 0.18 ± 0.03 × 10^12^ m^−1^, respectively), although the R_pb_ of the r-UF was still higher. Considering the pore sizes of the membranes used in the present study (0.4 µm for the c-MF membrane and 12 nm for the r-UF membrane), it was expected that the r-UF membrane would have presented a lower R_pb_. One possible explanation could be that the cake layer deposition that occurred quite quickly may provide a barrier (physical membrane/filter) for colloids and macromolecules that were supposed to participate in the pore-blocking process in both membranes. Nonetheless, smaller colloids and molecules could pass more easily through the cake layer and then participate in the blockage of smaller pores than the ones existing on UF membranes, as mentioned by Le-Clech et al., who observed that this tendency changes for long-term experiments [38]. Concerning, the reversibility of the membrane fouling layer, both membranes exhibited irreversible fouling mechanisms that accounted almost exclusively for the overall observed membrane fouling. Table 4 summarizes the contribution of the different fouling mechanisms to the observed fouling resistance. It was obvious that the irreversible fouling mechanisms were mainly responsible for the observed fouling phenomenon, thus making maintenance cleaning (mechanical and/or chemical) of both membranes necessary to restore their initial filtration performance.

Overall, it was observed that the relative contribution of each fouling mechanism was quite similar for c-MF and r-UF membranes. The resistance due to the irreversible fraction R_ir_ was the main resistance affecting *R_f_* in both cases. Furthermore, the r-UF membrane showed a lower relative R_pb_ contribution but higher relative R_c_. However, in both membranes, the cake layer remained the main fouling mechanism, accounting for approx. 60% of the overall fouling resistance.

### 3.3. Critical Factors Affecting Economic Sustainability

The sustainability of emerging technologies must be analyzed in the very early stages of their development. Ex-ante studies are more frequent and allow for the identification of trends and critical aspects to be resolved during the posterior research stages [39]. This section presents an analysis of the potential economic feasibility of the r-UF membranes. The analysis was performed in terms of two main units: (i) the cost of the production of 1 m^2^ of the r-UF membrane and (ii) the recycling cost of one module. Table 5 describes the results of the adaptation of the r-UF spiral wound module (originally a Toray TM-720 EoL-RO module) into five different commercial MBR flat sheet frames [26].

The different MBR frames were analyzed to allow for a membrane area recovery between 39% and 70%. The smaller frames allowed for a higher surface recovery due to the adaptation of the shape to the membrane sheet dimensions. However, the largest frames that are more common in the MBR membrane industry (with dimensions of 490 mm × 1000 mm) have a membrane area recovery above 60%. In all the cases, the cost is between 5.91–10.56 EUR·m^−2^, thus lower compared with commercial MBR prices (12.38–20.63 EUR·m^−2^) [40]. This lower cost could open the technological niche where the membrane replacement frequency is high or relatively higher, i.e., the treatment of harsh wastewaters or very intense processes where membranes are frequently damaged and replaced, such as for landfill leachate treatment. In this process, the membranes have a shorter expected lifespan (3–5 years), thus a high replacement ratio, representing 17% of the overall cost [41,42]. Data obtained in the present work showed that the permeability decline of the r-UF membrane would be slightly better under similar operating conditions (Table 3). Moreover, fouling analysis (Figure 7) showed that the r-UF membrane presented similar characteristics to the c-MF membrane. Therefore, the use of r-UF membranes could reduce the replacement cost. In the contrast, the r-UF is expected to have a higher energy cost due to the lower permeability. Membrane stability and low replacement cost could lead to an overall cost reduction of the process. Nonetheless, long-term tests should be performed to verify this hypothesis.

Besides the overall cost, the contribution analysis could identify crucial process steps (Table 6). The most expensive step in the process is the transformation of the whole module into the r-UF membrane and the logistics processes. These processes include, as shown in Figure 3, the transport processes (collection and distribution) and the chemical attack (with NaOCl) that transforms surface properties into an ultrafiltration membrane, as well as other downstream processes (washing and wastewater treatment). These costs were analyzed in detail in Senán-Salinas et al. [43]. However, within the new process steps, the main contributor was the manual disassembling, compromising 34% of the total cost of the treatment of one module (EUR 154 per module). This process needs to be done manually and its automatization through mechatronics engineering is challenging, although indispensable to reduce the cost of recycling. The same is true for the assembling and gluing of the sheets into new frames, which contributed approx. 7.4% to the overall cost. However, the sensitivity analysis, summarized in Table 7, identified the influence of changes in the main parameters on the overall cost. The sensitivity analysis pointed out the influence of the area recovered. Therefore, the development of a new frame with different sizes for the optimization of the area recovery could be useful for the scaling up.

One more factor that should be discussed concerning the potential implementation of r-UF membranes in MBR applications is the specific energy consumption (SEC) during the operation. SEC has been widely discussed and, for commercial MBRs, varies from 0.3 to 2.3 kWh·m^−3^ [45,46]. The most important factors affecting the SEC are the plant scale, the required permeate quality, and the blowers, as well as the operating conditions, including the net flow. As mentioned before, the permeability range is similar to other aMBR membranes with larger pore sizes (Figure 4) [47]. Therefore, the expected SEC can be similar. Furthermore, according to the literature, the contribution of permeate pumping is normally low compared with the overall aMBR processes (below 15%). Another critical factor is the permeability decline rate, ultimately leading to membrane chemical cleaning. As discussed in Section 3.2.2, the r-UF membrane presented a lower permeability decline rate over the whole experimentation period, during both 12 and 14 L·m^−2^·h^−1^ operation. It was reported in other studies that smaller membrane pore sizes are usually related to a slower TMP increase rate, thus resulting in longer membrane operation before chemical cleaning compared with larger pore membranes [48]. Even though the reported results in this study are preliminary and long-term test data are required to evaluate other main issues, such as the optimization of cleaning cycles and fouling dynamics, it seems that the r-UF membrane can perform comparably with the c-MF.

## 4. Conclusions

In the present work, a first reported proof-of-concept study to evaluate the feasibility of the use of recycled r-UF membranes as aMBR submerged membranes is provided. Overall, this study showed that the use of r-UF membranes in a flat sheet configuration in an aMBR system led to promising results. The TMP temporal evolution revealed that r-UF membranes exhibited a lower permeability decline rate, which may be beneficial for long-term working periods, whereas the fouling resistance analysis showed that the r-UF exhibited comparable characteristics to the widely employed c-MF membrane. However, the r-UF membrane permeability was much lower than MF membranes, which may negatively affect the cake layer fouling resistance due to the compaction of the cake. In terms of permeate quality, using the r-UF membrane, the laboratory-scale aMBR system exhibited excellent results regarding all studied parameters.

These encouraging results point to a very interesting alternative use of recycled EoL RO membranes in MBR systems and other UF processes where membrane replacement costs represent one of the main OPEX of the plant due to the elevated membrane replacement rate and/or important fouling issues. Although promising results were obtained, long-term experiments should be planned, including multiple experimental runs (replicates), especially to assess membrane fouling behavior in prolonged conditions. Moreover, planning new experimentation procedures at a larger scale should be considered. The collected data would be used for the performance of a detailed environmental sustainability assessment through life cycle analysis.

## Figures and Tables

**Table 1:** Commercial microfiltration (c-MF) and recycled ultrafiltration (r-UF) membranes’ technical data.

	Membrane Material	Nominal Permeability (20 °C)	Nominal Pore Size	Effective Membrane Area	R_a_	R_q_	Contact Angle
c-MF	Chlorinated polyethylene	1300 L·m^−2^·h^−1^·bar^−1^	0.4 µm	0.11 m^2^	184 ± 21 nm	234 ± 26 nm	104° [16]
r-UF	PES	255 L·m^−2^·h^−1^·bar^−1^	12 nm	0.11 m^2^	4.7 ± 0.6 nm [17]	6.3 ± 1.2 nm [17]	68° [15]

**Table 2:** Permeate quality and removal efficiency average values of the four different operating stages (Ia, Ib, IIa, and IIb). The *p*-values marked as * indicate the ANOVA results between permeate quality of the two membranes for every stage that were statistically significant with a confidence level over 95%.

	**Permeate Quality**		**Removal (%)**		***p*-Value**
	**(Ia) c-MF**	**(II a) r-UF**	**(Ia) c-MF**	**(II a) r-UF**	**r-UF-c-MF**
Turbidity (NTU)	0.14 ± 0.01	0.04 ± 0.02	-	-	0.000246 *
TOC (mg/L)	3.00 ± 0.26	1.82 ± 0.12	98.2 ± 0.2	98.9 ± 0.1	0.0006 *
Total N (mg/L)	26.01 ± 1.79	24.23 ± 2.59	17.2 ± 5.7	22.85 ± 9.5	0.40
Total P (mg/L)	3.68 ± 0.29	3.26 ± 0.65	29.9 ± 5.4	37.9 ± 14.2	0.50
COD (mg/L)	5.05 ± 0.64	5.93 ± 0.88	99.1 ± 0.2	98.8 ± 0.3	0.216
BOD_5_ (mg/L)	1.25 ± 0.35	<1	99.5 ± 0.1	99.7 ± 0.1	0.293
	**Permeate Quality**		**Removal (%)**		***p*-Value**
	**(Ib) c-MF**	**(II b) r-UF**	**(Ib) c-MF**	**(II b) r-UF**	**r-UF-c-MF**
Turbidity (NTU)	0.29 ± 0.32	0.01 ± 0.05	-	-	0.0919
TOC (mg/L)	2.28 ± 0.38	1.57 ± 0.20	98.6 ± 0.2	99.0 ± 0.1	0.01 *
Total N (mg/L)	22.21 ± 3.56	17.40 ± 6.34	29.3 ± 11.3	51.2 ± 14.3	0.02 *
Total P (mg/L)	3.58 ± 0.75	3.43 ± 0.31	31.8 ± 14.3	38.6 ± 6.6	0.39
COD (mg/L)	7.97 ± 1.73	4.52 ± 1.34	98.3 ± 0.4	98.9 ± 0.3	0.05
BOD_5_ (mg/L)	<1	<1	99.7 ± 0.1	99.6 ± 0.0	1

**Table 3:** Summary of membrane permeability decline values for the different operating stages.

Membrane	Flux (L·m^−2^·h^−1^)	Data Series	Permeability Decline Rate (L·m^−2^·h^−1^·bar^−1^·d^−1^)	R^2^	*p*-Value
c-MF	12	Days 1–7	43.9 ± 7.9	0.864	0.0025
c-MF	14	Days 8–22	51.6 ± 4.3	0.941	7.70 × 10^−7^
r-UF	12	Days 1–10	5.3 ± 0.7	0.835	0.00022
r-UF	14	Days 11–19	15.8 ± 1.9	0.911	6.35 × 10^−5^

**Table 4:** Relative contribution (%) of different membrane fouling mechanisms for the c-MF and r-UF membranes after stages Ib and IIb.

Membrane Type	Membrane Fouling Mechanisms			
	Cake Layer	Pore Blocking	Reversible	Irreversible
c-MF	58.6 ± 16.6%	41.4 ± 6.1%	8.5 ± 8.5%	91.5 ± 14.1%
r-UF	66.4 ± 4.4%	33.6 ± 1.9%	0.0 ± 2.5%	100.0 ± 3.8%

**Table 5:** Cost of the production of the r-UF for use in an aMBR depending on the targeted membrane frame size.

Commercial Membrane Modules/Frames	Sheet Dimensions (mm)	Number of Sheets Cut	Area Recovered (m^2^)	Area Recovered per Module (%)	Cost (EUR·m^−2^)
Recycled Toray TM 720	960 × 845	1	37	-	-
Kubota-510 SINAP-80	490 × 1000	1	22.2	60	6.89
Kubota-203	226 × 316	8	25.9	70	5.91
SINAP-25	340 × 470	2	14.4	39	10.56
SINAP-10	220 × 320	8	25.5	69	5.99

**Table 6:** Cost contribution analysis of the different processes for r-UF membrane preparation.

Cost Type	Process	Cost per Module (EUR·Module^−1^)	Cost Contribution	Source
CAPEX + OPEX	Module transformation to the r-UF membrane, characterization, and logistics	80	51.96%	[7]
OPEX-Labor	Disassembling and sheet cutting	51.17	33.24%	Own data
OPEX-Labor	Re-assembling in new frames	11.37	7.39%	Own data
OPEX-Energy	Electricity use during the processing	0.03	0.02%	[44]
Total cost	Recycling of one module	153.95	100%	

**Table 7:** Sensitivity analysis results of the principal parameters affecting the r-UF membrane production cost.

Parameter	Effect (Δ%)	Ratio (Δ% Effect/Δ% Parameter)
Reduce 25% of area recovered	33	1.32
Change 25% of transformation cost	13	0.52
Change 25% of labor cost	12	0.48

## Data Availability

Not applicable.

## Abbreviations

aMBR	Aerobic membrane bioreactor	MWCO	Molecular weight cut-off
BOD_5_	Biological oxygen demand	NF	Nanofiltration
CAPEX	Capital expenditure	OPEX	Operational expenditure
CAS	Conventional activated sludge	PA	Polyamide
c−MF	Commercial microfiltration	PES	Polyethersulfone
COD	Chemical oxygen demand	PSF	Polysulfone
cSWW	Concentrated synthetic wastewater	RO	Reverse osmosis
DO	Dissolved oxygen	r-UF	Recycled ultrafiltration
EoL	End of life	SEC	Specific energy consumption
HRT	Hydraulic retention time	SRT	Solid retention time
MBR	Membrane bioreactor	TN	Total nitrogen
MF	Microfiltration	TOC	Total organic carbon
MLSS	Mixed liquor suspended solids	TP	Total phosphorous
L	Membrane permeability at (L·m^− 2^·h^−1^·bar^−1^)	R_m_	Membrane resistance (m^−1^)
L_S_	Membrane permeability at a certain time (L·m^−2^·h^−1^·bar^−1^)	R_pb_	Pore blocking resistance (m^−1^)
J	Flux (L·m^−2^·h^−1^)	R_q_	Root mean square average of height deviation (nm)
R	Resistance (m^−1^)	R_rev_	Reversible resistance (m^−1^)
R_a_	Arithmetic average of absolute values of surface height deviations (nm)	R_T_	Total resistance (m^−1^)
R_c_	Cake layer resistance (m^−1^)	t	Time (h)
R_c(ir)_	Cake layer irreversible resistance (m^−1^)	T	Temperature (°C)
R_c(rev)_	Cake layer reversible resistance (m^−1^)	TMP	Transmembrane pressure (Pa)
R_f_	Fouling resistance (m^−1^)	µ	Dynamic viscosity (Pa·s)
R_ir_	Irreversible resistance (m^−1^)		
